# Complete genome sequences of three *Brucella abortus* strains isolated from animals in 1954 in Switzerland

**DOI:** 10.1128/mra.00250-26

**Published:** 2026-06-15

**Authors:** Ezgi Akdesir, Loïc Borcard, Alban Ramette

**Affiliations:** 1 Department of Infectious Diseases and Pathobiology, Vetsuisse Faculty, Institute of Veterinary Bacteriology, University of Bern 27210, Bern, Switzerland; 2 Institute for Infectious Diseases, University of Bern 27210, Bern, Switzerland; 3 Multidisciplinary Center for Infectious Diseases, University of Bern 27210, Bern, Switzerland; Portland State University, Portland, Oregon, USA

**Keywords:** zoonosis, brucellosis, ibex, roe deer, cow

## Abstract

Identification of *Brucella* species based on the phenotype can be challenging, and it is important to apply genome-based methods for correct identification of these species and for understanding the dynamics of this important zoonosis. Genomes of three *Brucella* strains archived in 1954 acquired from animals in Switzerland are presented.

## ANNOUNCEMENT

Brucellosis is a major zoonotic disease, and due to its chronicity and non-specific symptoms in humans, it may progress to severe disease due to delayed diagnosis (1–3). The close genetic relationship among *Brucella* species presents a diagnostic challenge (4). The main species affecting animals are *B. abortus*, *B. melitensis*, *B. suis*, and *B. canis*, with *B. melitensis* infecting humans most frequently.

Here, we present the complete genome sequences (Table 1) of three field isolates archived from Switzerland, which were isolated from an ibex (strain 36504), a roe deer (strain 57324), and a cow (strain 64425) in 1954. These isolates were then identified as *B. abortus* based on biochemical tests (5); however, it was not possible to determine their genetic similarity in the pre-genomics era.

**TABLE 1:** Summary statistics for the hybrid genome assemblies of each strain^*a*^

Strains	Input reads (M reads/average read size)		Assembly quality parameters		
	ONT	Illumina	N50 (Kbp)	Length (bp)	Completeness (%)
36504	0.9 M/2,023.9 bases	1.0 M/150 bases	2,125.6	3,285,814	98.8
57324	2.3 M/2,431.7 bases	0.8 M/150 bases	2,124.1	3,285,705	98.8
64425	0.9 M/2,286.3 bases	0.7 M/150 bases	2,125.3	3,286,828	98.9

^*a*^ The input reads correspond to all the reads used for each technology as input to generate the genome assemblies. Assembly quality parameters were provided by QUAST and BUSCO. Each assembled genome consisted of two circular chromosomes of size 2.12 and 1.16 Mbp.

Lyophilized isolates, kept at +4°C, were resuspended in sterile PBS and grown on Columbia agar with 5% sheep blood (citrated sheep blood agar [CSBA]; Oxoid Thermo Fisher Scientific, Switzerland) at 35°C aerobically with 5% CO_2_ supplementation. Genomic DNA was isolated from heat-inactivated colonies using the PureLink Genomic DNA kit (Thermo Fisher Scientific). Illumina sequencing libraries were produced with the Nextera DNA Flex Library Prep kit (Illumina, Switzerland) and sequenced on an Illumina MiSeq sequencer (v2; 300 cycles; paired-end mode). For Oxford Nanopore Technologies (ONT), each sample’s concentration was normalized to 20 ng/L, and sequencing libraries were produced with the rapid barcoding chemistry SQK-RBK114.96 (RBK) using the standard protocol provided by the manufacturer and sequenced according to ONT recommendations on standard GridION flowcells (FLO-MIN114) under high accuracy basecalling mode (“HAC,” model version dna_r10.4.1_e8.2_400bps_hac@v4.3.0) using Dorado (v7.6.7) and MinKNOW (v6.2.6; default parameters). Raw sequencing data from both sequencing technologies were processed together to generate hybrid assemblies with the pipeline nf-core/bacass (version 2.5.0, https://nf-co.re/bacass/2.5.0/; using dragonflye v1.2.1 [6, 7]; fastp v0.24.0 [8]; FastQC v0.12.1 [9]; medaka v1.4.3 [10]; Porechop v0.2.4 [6]; QUAST v5.2.0 [11]; toulligQC v2.7.1 [12], Prokka v1.14.6 [13]). Completeness was assessed using BUSCO v6.0.0 (14) using the hyphomicrobiales_odb12 data set. Default software parameters were used in all applications, if not specified otherwise.

**Fig 1:**
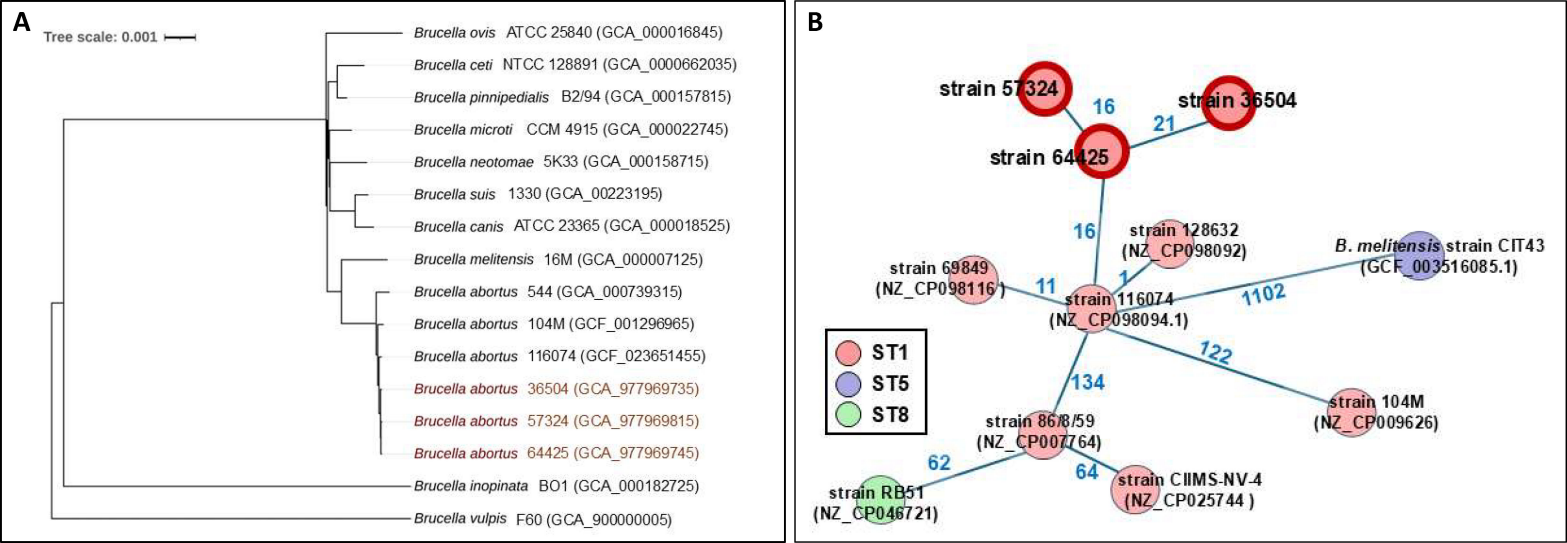
(**A**) Phylogram generated in Type (Strain) Genome Server (15) and the LPSN database (16, 17), including the strains of the study highlighted in red (strain: 36504, strain: 57324, strain: 64425 from an ibex, roe deer, and cow respectively), closest neighbor strain (*B. abortus* strain 116074) based on cgMLST analysis, a vaccine strain (*B. abortus* 104M), and other reference *Brucella* sp. strains. The tree was inferred with FastME v2.1.6.1 (22) from GBDP (Genome BLAST Distance Phylogeny) distances calculated from genome sequences (9). The branch lengths are scaled in terms of GBDP distance formula d5. The tree was rooted at the midpoint. (**B**) Minimum spanning tree (MST) representation of the allelic distances between ten *Brucella abortus* and one *B. melitensis* genomic sequences using SeqSphere+. The three strains of the study are highlighted in red circles. The allelic profiles of MLST and cgMLST are based on *Brucella* sp. task template v1.0 with 9 and 1,764 loci (pairwise ignoring missing values), respectively. Allelic distances are indicated along the edges, and nodes are colored by ST.

The read GC content was 57.2% for all three isolates. Their genomes consisted of two circular chromosomes (Table 1). The assemblies contained 3,119 to 3,126 coding sequences (CDS), and all harbored 9 rRNA, 55 tRNA, and 1 tmRNA genes. Whole genome-based taxonomic analysis identified all isolates as *B. abortus* (Fig. 1A) using Type (Strain) Genome Server (https://tygs.dsmz.de [15]) and the LPSN database (https://lpsn.dsmz.de) (16, 17). All three isolates belonged to sequence type 1 (ST1) based on multi-locus sequence typing (MLST) with MLST 2.0 tool of the Center for Genomic Epidemiology (10). All three isolates cluster closely together but do not belong to the same clonal complex based on core genome MLST (cgMLST) analysis (Task template “Brucella abortus,” v1.0 (18); SeqSphere+; v10.0.0; Ridom GmbH, Germany), given pairwise genetic distances of 16–24 allelic differences (18) (Fig. 1B). The core genome was defined using Roary (v3.13.0 [19]), resulting in 3,105 shared genes. These genes were aligned with MAFFT (v7.508 [20]) and pairwise genetic distances (number of nucleotide differences) between genomes were subsequently calculated in MEGA X (v10.1.8 [21]), yielding 37–39 core SNP differences. *B. abortus* strain 116074 isolated from a water buffalo in the Campania Region of Italy is the closest neighbor of the study isolates.

## Data Availability

Complete genome sequences of *B. abortus* strains 36504, 57324, and 64425 have been deposited in the European Nucleotide Archive (ENA) under BioProject PRJEB105433, at ENA accession numbers GCA_977969735, GCA_977969815, and GCA_977969745, respectively. FASTQ reads (Illumina, ONT) are available at ENA accession numbers (ERR16037262, ERR16037903), (ERR16037264, ERR16037906), and (ERR16037265, ERR16037929), respectively.
